# Reversibility of delirium in Ill‐hospitalized cancer patients: Does underlying etiology matter?

**DOI:** 10.1002/cam4.2669

**Published:** 2019-11-06

**Authors:** Yoshinobu Matsuda, Isseki Maeda, Tatsuya Morita, Toshihiro Yamauchi, Akihiro Sakashita, Hiroaki Watanabe, Keisuke Kaneishi, Koji Amano, Satoru Iwase, Asao Ogawa, Kazuhiro Yoshiuchi, on behalf of the Phase‐R Delirium Study Group, Hirofumi Abo, Tatsuo Akechi, Nobuya Akizuki, Toru Okuyama, Daisuke Fujisawa, Shingo Hagiwara, Takeshi Hirohashi, Takayuki Hisanaga, Kengo Imai, Shuji Inada, Satoshi Inoue, Shinichiro Inoue, Aio Iwata, Akifumi Kumano, Takashi Matsui, Yoshihisa Matsumoto, Naoki Matsuo, Kaya Miyajima, Ichiro Mori, Sachiyo Morita, Rika Nakahara, Nobuhisa Nakajima, Hiroyuki Nobata, Takuya Odagiri, Ken Shimizu, Yuki Sumazaki Watanabe, Keita Tagami, Emi Takeuchi, Mari Takeuchi, Ryohei Tatara, Akihiro Tokoro, Megumi Uchida, Keiichi Uemura, Ritsuko Yabuki, Naosuke Yokomichi

**Affiliations:** ^1^ Department of Psychosomatic Internal Medicine National Hospital Organization Kinki‐Chuo Chest Medical Center Osaka Japan; ^2^ Gratia Hospice Gratia Hospital Osaka Japan; ^3^ Palliative and Supportive Care Division Seirei Mikatahara General Hospital Shizuoka Japan; ^4^ Seirei Hospice Seirei Mikatahara General Hospital Shizuoka Japan; ^5^ Department of Palliative Medicine Kobe University Graduate School of Medicine Hyogo Japan; ^6^ Department of Palliative Care Komaki City Hospital Aichi Japan; ^7^ Department of Palliative Care Unit JCHO Tokyo Shinjuku Medical Center Tokyo Japan; ^8^ Department of Palliative Medicine Osaka City General Hospital Osaka Japan; ^9^ Department of Palliative Medicine Saitama Medical University Saitama Japan; ^10^ Department of Psycho‐Oncology Exploratory Oncology Research and Clinical Trial Center National Cancer Center Chiba Japan; ^11^ Department of Stress Science and Psychosomatic Medicine Graduate School of Medicine The University of Tokyo Tokyo Japan

**Keywords:** cancer, cause, delirium, palliative care, reversibility

## Abstract

**Background:**

The objective of this study was to explore the underlying etiologies associated with the resolution and improvement of delirium in ill‐hospitalized cancer patients.

**Methods:**

We conducted a secondary analysis of a multicenter, prospective, observational study to estimate the effectiveness of pharmacotherapy for delirium. Participants were cancer patients with delirium. We assessed the Delirium Rating Scale, Revised‐98 (DRS‐R98) severity scale score at baseline and three days after pharmacotherapy initiation. Delirium resolution was defined as a DRS‐R98 severity scale score ≤9, and improvement was defined as ≥50% reduction at Day 3.

**Results:**

We enrolled 566 patients (491 patients had performance status of 3 or 4). The resolution and improvement rates in all patients were 22.6% and 19.3%, respectively. Univariate analysis determined that nonrespiratory infection (OR 2.18, 95% CI 1.38‐3.45) was significantly associated with greater resolution, while dehydration (0.40, 0.19‐0.87), organic damage to the central nervous system (CNS) (0.32, 0.43‐0.72), hypoxia (0.25, 0.12‐0.52), and hyponatremia (0.34, 0.12‐0.97) were significantly associated with no resolution. Potential causes associated with delirium improvement were nonrespiratory infection (1.93, 1.19‐3.13), organic damage to the CNS (0.40, 0.18‐1.90), and hypoxia (0.32, 0.16‐0.65). After multivariate analysis, dehydration (0.34, 0.15‐0.76), organic damage to the CNS (0.25, 0.10‐0.60), and hypoxia (0.29, 0.14‐0.61) were significantly associated with no resolution.

**Conclusions:**

Delirium caused by nonrespiratory infection may be reversible, while delirium associated with dehydration, organic damage to the CNS, hypoxia, or hyponatremia seems to be irreversible in ill‐hospitalized cancer patients.

## Graphical abstract

We confirmed that delirium caused by nonrespiratory infection can be reversible, while delirium associated with dehydration, organic damage to the central nervous system, hypoxia, and hyponatremia is likely irreversible.

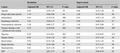

## 1 INTRODUCTION

Delirium is a common symptom in patients with cancer^1^, ^2^, ^3^, ^4^ and causes distress to both the affected patients and their families.^5^, ^6^ Delirium is associated with increased morbidity and mortality, and higher healthcare costs.^7^, ^8^ Thus, it is important to manage delirium in patients with cancer. Antipsychotics have been used to manage the symptoms of delirium in cancer patients.^9^, ^10^, ^11^, ^12^ However, a randomized clinical trial comparing antipsychotics to placebo in patients receiving palliative care revealed that the delirium symptom score was higher in the antipsychotic groups than in the placebo group, suggesting that management of the causes for delirium and supportive strategies may be more effective than administering antipsychotics.^13^ Opioids, hypnotics, anxiolytics, corticosteroids, anticholinergic drugs, hypercalcemia, hyponatremia, dehydration, hypoxia, infection, and organic damage to the central nervous system (CNS) have been reported as causes of delirium.^14^, ^15^, ^16^, ^17^, ^18^ One standard treatment for delirium is to identify and manage such underlying causes.^17^ Knowledge of the reversibility of various causes of delirium is an important consideration in planning suitable treatment strategies for individual patients. Only a few small studies, however, have reported on the causes that may be reversible.^1^, ^19^

The objective of this study was to identify the causes associated with the resolution and improvement of delirium in cancer patients using data from an existing study of delirium in cancer patients (Japan Pharmacological Audit study of Safety and Efficacy in Real world; Phase‐R).

## 2 METHODS

The present study is a secondary analysis of a multicenter, prospective, observational study that primarily aimed to estimate the effectiveness and adverse events of pharmacotherapy in cancer patients. Patients were enrolled from September 2015 to May 2016.

This study was approved by the Institutional Review Boards of all participating sites (approval number #13295 in Osaka University). Informed consent was not obtained in this study because we observed usual clinical practice including treatment and assessments. We used an opt‐out method so that patients and families could refuse to participate in the study.

### 2.1 Study setting and Subjects

The participating sites were 14 palliative care units and 9 psycho‐oncology liaison services situated across Japan.

Patients eligible for the original study were (a) cancer patients with delirium diagnosed according to the Diagnostic and Statistical Manual of Mental Disorders, Fifth Edition by the treating palliative care physicians or psycho‐oncologists^20^ and (b) patients who would receive antipsychotics or trazodone for delirium. Trazodone was included in pharmacotherapy because trazodone is often prescribed for delirium in Japan.^21^, ^22^ Patients were excluded if (a) patients or their family refused to participate in the study, (b) patients had postoperative delirium, or (c) patients had alcohol‐ or drug‐withdrawal delirium.

### 2.2 Procedure and measurements

Patients who met the eligibility criteria were enrolled in the original study at any phase of hospitalization. The dose and type of pharmacotherapy were prescribed by the palliative care physician or psycho‐oncologist according to their typical clinical practice. No specific treatment guidelines were used.

To determine the severity of the delirium, we assessed it using the Delirium Rating Scale, Revised‐98 (DRS‐R98) severity scale at baseline and three days after the initiation of pharmacotherapy. Day 3 was selected for evaluation, based on the poor prognosis for our population and previous studies suggesting that a rough estimation of the treatment effectiveness is possible at this time point.^10^, ^23^ We used baseline patient data including patient age, sex, Eastern Cooperative Oncology Group (ECOG) Performance Status,^24^ the Palliative Performance Scale score,^25^ clinical prediction of survival (days, weeks, and months), the Palliative Prognostic Index,^26^ delirium subtypes, primary tumor site information, and the potential causes of delirium. Delirium subtypes were assessed using the Delirium Motor Subtype Scale.^27^, ^28^ In this scale, the delirium is categorized as the hyperactive subtype when a patient has at least two of four symptoms, such as restlessness and increased quantity of motor activity. The delirium is categorized as the hypoactive subtype when a patient has two or more of seven symptoms, such as decreased amount of activity.

The treating palliative care physicians or psycho‐oncologists were also asked to suggest potential causes of the delirium based on their clinical judgement and according to medical histories, physical examinations, and laboratory or radiological findings. They were asked to select the potential causes from a list we prepared with reference to a previous study.^1^ There were no operational criteria for each etiology. We categorized infection into respiratory or nonrespiratory, based on the assumption that the former could be associated with hypoxia and irreversibility. We judged that a factor contributed to delirium if (a) a specific pathological feature known to cause delirium was identified and (b) there was a temporal association between the delirium onset and the occurrence of the pathological feature (ie, the feature precedes delirium onset).^19^ Changes in delirium severity in association with changes in the cause after pharmacotherapy for delirium were not included in the definition of the causes. This is because in this study setting, blood sampling for the follow‐up presented practical difficulties in many cases.

### 2.3 DRS‐R98 severity scale

The DRS‐R98 is a 16‐item clinician‐rated scale with 13 severity items and 3 diagnostic items. The range of each severity item is 0 to 3, and the range of the total score is 0 to 39, with a higher score indicating more severe delirium. This scale has high reliability and validity in its original language.^29^ We used the Japanese version of the DRS‐R98, which has sufficient reliability and validity.^30^

### 2.4 Statistical analysis

For analyses, we used the patients who were treated with first‐line pharmacotherapy and patients with DRS‐R98 severity scale scores of ≥10 at baseline. The value of ≥10 was chosen because the cutoff score for the diagnosis of delirium in the Japanese version of the DRS‐R98 severity scale score is 9/10.^30^

Resolution of delirium was defined as a reduction in the DRS‐R98 severity scale score from ≥10 at baseline to a score of ≤9 at Day 3. Improvement of delirium was defined as ≥a 50% reduction in the DRS‐R98 severity scale score at Day 3 compared with baseline, in accordance with previous studies.^9^, ^31^ We decided not to evaluate the changes in mean values of the DRS‐R98 severity scale score, because there is no consensus how we can interpret the changes in mean scores when the patients were dying.^32^

For patients that experienced a decline in the consciousness (ie, Richmond Agitation and Sedation Scale by 3 or more points) or patients who died or ceased pharmacotherapy due to general deterioration or noneffectiveness before Day 3, the following DRS‐R98 severity scale items at Day 3 were scored as 3 (denoting the highest severity): sleep‐wake cycle disturbance, language, thought process abnormalities, motor retardation, orientation, attention, short‐term memory, long‐term memory, and visuospatial ability. This approach was taken in accordance with a previous study.^30^ Odds ratios with 95% confidence intervals were calculated for the resolution and improvement rates for each cause of delirium. Univariate analyses were performed using the chi‐square test. Multivariate analyses were performed using logistic regression with the causes of delirium, age, ECOG Performance status (0, 1, 2, 3, or 4), prognosis estimation (days, weeks, or months), and setting (palliative care units or psycho‐oncology liaison services) as the independent factors, and resolution or improvement of delirium as the dependent factor. P‐values less than 0.05 were regarded as statistically significant. All analyses were performed using SPSS software, version 23 (SPSS Inc).

## 3 RESULTS

### 3.1 Patient characteristics

A total of 702 patients were enrolled in the original study. Five patients were excluded because their DRS‐R98 severity scale score at Day 3 was missing (n = 1 discharged; n = 2 ineligible after enrollment; n = 2 unknown). Of the remaining 697 patients, 131 patients were further excluded because their DRS‐R98 severity scale scores at baseline were ≤9. The 566 patients were included in this study. By Day 3, 157 patients had lost consciousness, 10 had expired, and 2 had not received any pharmacotherapy due to general deterioration (n = 1) or noneffectiveness (n = 1). Most patients (87%) had ECOG Performance Status 3 or 4, and about 60% of patients were enrolled from palliative care units (Table 1).

**Table 1:** Patient characteristics

Number of patients	566
Sex, male (%)	351 (62.0)
Age, mean (SD), y	72.0 (11.3)
ECOG performance status, n (%)	
0, 1, 2	75 (13.3)
3	217 (38.3)
4	274 (48.4)
Palliative performance scale, n (%)	
60‐	25 (4.4)
30‐50	275 (48.6)
10‐20	266 (47.0)
Palliative prognostic index, n (%)	
≥6.5	543 (95.9)
<6.5	23 (4.1)
Prognosis estimation, n (%)	
Days	109 (19.3)
Weeks	272 (48.1)
Months	185 (32.7)
Delirium subtypes	
Hyperactive subtype, n (%)	197 (34.8)
Mixed motor subtype, n (%)	202 (35.7)
Hypoactive subtype, n (%)	167 (29.5)
Setting, n (%)	
Consultation with psycho‐oncologists in general hospitals	203 (35.9)
Palliative care units	363 (64.1)
Primary tumor sites, n (%)	
Lung	128 (22.6)
Esophagus	18 (3.2)
Stomach	53 (9.4)
Colon/rectum	61 (10.8)
Liver/biliary system/pancreas	95 (16.8)
Breast	30 (5.3)
Kidney/bladder/urinary tract/prostate	53 (9.4)
Uterine/ovary	33 (5.8)
Head and neck	29 (5.1)
Blood/lymph node	22 (3.9)
Brain	5 (0.9)
Others	39 (6.9)

Abbreviations: ECOG, Eastern Cooperative Oncology Group.

### 3.2 Resolution and improvement of delirium

**Figure 1:**
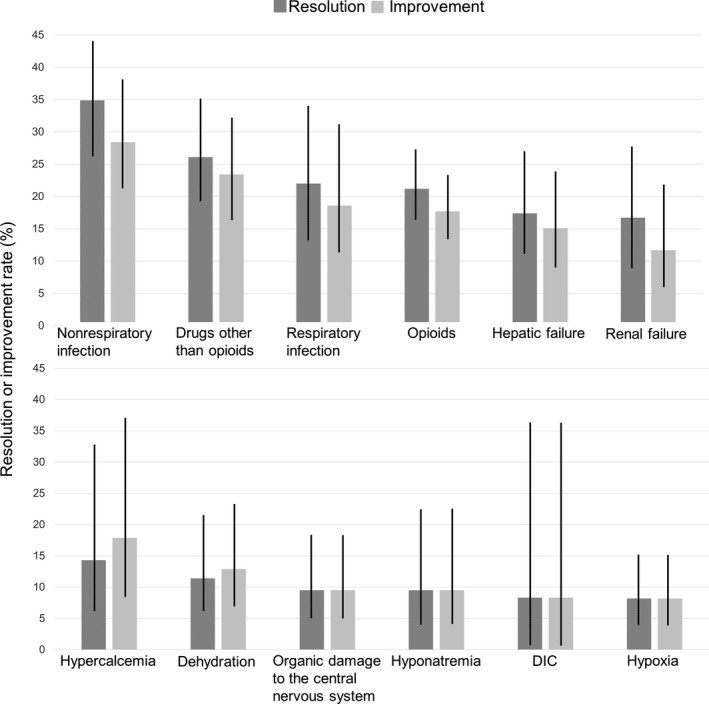
Resolution or improvement rates of the causes of delirium. Abbreviations: DIC, disseminated intravascular coagulation. Error bars indicate 95% confidence intervals

The resolution and improvement rates in all patients were 22.6% and 19.3%, respectively. The highest resolution rate was observed with nonrespiratory infection (34.9%), followed by drugs other than opioids (26.1%), respiratory infection (22.0%), and opioids (21.2%). The lowest resolution rate was observed with hypoxia (8.2%), followed by disseminated intravascular coagulation (8.3%), hyponatremia (9.5%), and organic damage to the CNS (9.5%) (Figure 1).

Univariate analysis determined that nonrespiratory infection (OR 2.18, 95% CI 1.38‐3.45) was significantly associated with greater resolution in the DRS‐R98 severity scale score, while dehydration (OR 0.40, 95% CI 0.19‐0.87), organic damage to the CNS (OR 0.32, 95% CI 0.43‐0.72), hypoxia (OR 0.25, 95% CI 0.12‐0.52), and hyponatremia (OR 0.34, 95% CI 0.12‐0.97) were significantly associated with no resolution. Potential causes associated with delirium improvement were nonrespiratory infection (OR 1.93, 95% CI 1.19‐3.13), organic damage to the CNS (OR 0.40, 95% CI 0.18‐1.90), and hypoxia (OR 0.32, 95% CI 0.16‐0.65) (Table 2).

**Table 2:** Univariate analysis of the causes associated with the resolution and improvement of delirium

Causes	Presence n (%)	Resolution		Improvement	
		OR (95% CI)	*P*‐value	OR (95% CI)	*P*‐value
Opioids	231 (40.8)	0.87 (0.58‐1.31)	.51	0.85 (0.55‐1.30)	.45
Drugs other than opioids	111 (19.6)	1.27 (0.79‐2.05)	.32	1.37 (0.83‐2.26)	.21
Dehydration	70 (12.4)	0.40 (0.19‐0.87)	.017	0.58 (0.28‐1.22)	.147
Respiratory infection	59 (10.4)	0.96 (0.50‐1.85)	.91	0.96 (0.48‐1.90)	.90
Nonrespiratory infection	109 (19.3)	2.18 (1.38‐3.45)	.001	1.93 (1.19‐3.13)	.007
Organic damage to the central nervous system	74 (13.1)	0.32 (0.43‐0.72)	.004	0.40 (0.18‐1.90)	.022
Hypoxia	110 (19.4)	0.25 (0.12‐0.52)	<.001	0.32 (0.16‐0.65)	.001
Hepatic failure	86 (15.2)	0.69 (0.38‐1.25)	.21	0.71 (0.38‐1.34)	.29
Renal failure	60 (10.6)	0.66 (0.32‐1.34)	.24	0.52 (0.23‐1.19)	.12
Hypercalcemia	28 (4.9)	0.56 (0.19‐1.63)	.28	0.91 (0.34‐2.44)	.85
Hyponatremia	42 (7.4)	0.34 (0.12‐0.97)	.035	0.42 (0.15‐1.20)	.10
DIC	12 (2.1)	0.31 (0.04‐2.39)	.23	0.38 (0.05‐2.94)	.33

Abbreviations: CI, confidence interval; DIC, disseminated intravascular coagulation; OR, odds ratio.

In multivariate analysis, dehydration (OR 0.34, 95% CI 0.15‐0.76), organic damage to the CNS (OR 0.25, 95% CI 0.10‐0.60), and hypoxia (OR 0.29, 95% CI 0.14‐0.61) were significantly associated with no resolution of delirium. Organic damage to the CNS (OR 0.28, 95% CI 0.12‐0.69) and hypoxia (OR 0.39, 95% CI 0.19‐0.83) were significantly associated with no improvement of delirium. (Table 3).

**Table 3:** Multivariate analysis of the causes associated with the response and resolution of delirium

Causes	Resolution			Improvement		
	Adjusted OR	95% CI	*P*‐value	Adjusted OR	95% CI	*P*‐value
Opioids	0.94	0.59‐1.49	.79	0.86	0.53‐1.40	.54
Drugs other than opioids	1.19	0.68‐2.08	.54	1.28	0.72‐2.28	.40
Dehydration	0.34	0.15‐0.76	.009	0.54	0.25‐1.19	.129
Respiratory infection	1.01	0.48‐2.13	.98	0.99	0.46‐2.14	.97
Nonrespiratory infection	1.38	0.80‐2.37	.25	1.18	0.67‐2.06	.57
Organic damage to the central nervous system	0.25	0.10‐0.60	.002	0.28	0.12‐0.69	.005
Hypoxia	0.29	0.14‐0.61	.001	0.39	0.19‐0.83	.015
Hepatic failure	0.65	0.34‐1.25	.165	0.73	0.37‐1.44	.36
Renal failure	0.76	0.35‐1.66	.49	0.59	0.25‐1.42	.24
Hypercalcemia	0.59	0.19‐1.86	.36	1.07	0.37‐3.13	.90
Hyponatremia	0.42	0.14‐1.30	.13	0.54	0.17‐1.65	.28
DIC	0.57	0.07‐4.74	.60	0.78	0.09‐6.49	.82

Abbreviations: CI, confidence interval; DIC, disseminated intravascular coagulation; OR, odds ratio.

## 4 DISCUSSION

This is one of the largest study evaluating the potential causes associated with the resolution and improvement of delirium in ill‐hospitalized cancer patients. Univariate analysis identified nonrespiratory infection as associated with significantly greater resolution in the DRS‐R98 severity scale score, whereas dehydration, organic damage to the CNS, hypoxia, and hyponatremia were associated with significantly no resolution. Potential causes associated with a significant improvement of delirium were similar, except for dehydration and hyponatremia. After multivariate analysis, dehydration, organic damage to the CNS, and hypoxia were significantly associated with no resolution of delirium, while organic damage to the CNS and hypoxia were significantly associated with no improvement.

Our finding that organic damage to the CNS and hypoxia were significantly associated with no resolution and no improvement is in line with the results of the two previous studies.^1^, ^19^ Organic damage to the CNS and hypoxia seem to be associated with irreversibility.

Our study revealed that nonrespiratory infection was associated with greater resolution and improvement. However, one previous study found that nonrespiratory infection was associated with no improvement.^1^ Potential explanations include heterogeneity of nonrespiratory infections (eg, sepsis or urinary tract infections), inconsistent definition of infections, and small sample size. Further study is warranted to clarify the association between types of infection and the reversibility of delirium.

In the present study, delirium that was potentially caused by opioids was not significantly associated with greater resolution or improvement. Nevertheless, it has been associated with high reversibility in two previous studies.^1^, ^6^ A potential explanation for this inconsistency is as follows. Firstly, there are apparent differences in the general condition of patients. In the study conducted by Lawlor et al, the patients had a better performance status and were treated in an acute palliative care unit, and 20% of them did not have metastasis. It is reasonable to conclude that delirium caused by opioids was may be reversible in this situation. Secondly, the definition of the cause itself may have some effect on the findings. In the study by Lawlor et al, a positive treatment effect after dose reduction, discontinuation, or opioid switching was included in the criteria used to confirm opioids as the cause of the delirium. Thirdly, there may be differences across studies in the clinical practice of switching opioids in patients with delirium. In our study, the resolution rate of delirium caused by opioids was relatively high (21.2%) compared with other factors, although this difference was not statistically significant. These findings suggest that opioid switching or reducing the opioid dosage should be considered when treating opioid‐induced delirium, especially in patients in good general condition and those without other irreversible causes related to organ failure. The association between opioid usage and delirium reversibility should be investigated, ideally through interventional studies, using a homogeneous patient group, identical diagnostic criteria and intervention strategies, and specific opioid switching. In particular, the role of opioid switching in delirium in patients with poor general condition and organ failure would be of great value.

With respect to dehydration, the results differ between all three studies: Reversibility was high in the study by Lawlor et al^1^ but low in Morita et al^19^ and the present study. This inconsistency may be explained by the use of different criteria to define dehydration and differences in the general condition of the patients. In the present study, the prognosis estimation for almost 70% of patients was on the order of days or weeks, and almost 90% had performance status of 3 or 4. These findings were similar to those of Morita et al, whose study was conducted in an inpatient hospice. The patients in the study by Lawlor et al, however, had better performance status scores. A randomized clinical trial found that clinically assisted hydration is not effective for preventing delirium in hospice patients with moderate dehydration, but this finding is not applicable to the treatment of patients who already have delirium.^33^ Further clinical trials are therefore warranted to investigate the role of hydration treatment in managing delirium with dehydration, although it seems to be less reversible in ill‐hospitalized cancer patients.

This study is a secondary analysis and is characterized by considerable limitations. Firstly, it was not primarily designed to identify the causes associated with the resolution or improvement of delirium. In particular, we did not use operational criteria to determine whether an underlying etiology was associated with the development of delirium. Secondly, all patients in this study received pharmacotherapy treatment for delirium, so patients with mild‐to‐moderate delirium could not be enrolled. Thirdly, 30% of the cases studied were categorized as hypoactive delirium, although in general these patients would not be administered with pharmacotherapy. This discrepancy is probably because we used the Delirium Motor Subtype Scale to classify the delirium. With this scale, patients with symptoms meeting the criteria of the hypoactive subtype would be classified as such, even if they also had one symptom of the hyperactive subtype. This may have influenced the low reversibility observed in our results.^34^ Fourthly, we assessed the reversibility of delirium at Day 3, although we acknowledge that this time frame may not be sufficient for recovery from delirium. Thus, we may have underestimated the reversibility of delirium in this study. Finally, our findings are not generalizable to cancer patients with good general condition, since 90% of patients in this study had a performance status of 3 or 4.

In conclusion, delirium caused by nonrespiratory infection may be reversible, whereas delirium associated with dehydration, organic damage to the CNS, hypoxia, or hyponatremia seems to be irreversible in ill‐hospitalized cancer patients. The effect of infection, opioids, and dehydration on the reversibility of delirium requires further investigation in studies (ideally clinical trials) involving a homogeneous population and using standard criteria. Finally, the development of a comprehensive prediction tool using not only potential causes but also a wide range of information, and not only potential causes, to identify reversible delirium would be highly valuable for the treatment of ill‐hospitalized cancer patients.

## CONFLICT OF INTEREST

All authors declare no conflicts of interest.

## AUTHOR CONTRIBUTIONS

Yoshinobu Matsuda: planned, wrote, and revised the writing and concept of the article, and analyzed data. Isseki Maeda: planned the concept of the article and reviewed and edited the article. Tatsuya Morita: planned the concept of the article and reviewed and edited the article. Toshihiro Yamauchi: reviewed and edited the article. Akihiro Sakashita: reviewed and edited the article. Hiroaki Watanabe: reviewed and edited the article. Keisuke Kaneishi: reviewed and edited the article. Koji Amano: reviewed and edited the article. Satoru Iwase: reviewed and edited the article. Asao Ogawa: reviewed and edited the article. Kazuhiro Yoshiuchi: reviewed and edited the article.

## APPENDIX 1

Collaborators of the Phase‐R Delirium Study Group (in addition to the authors listed above) (alphabetical order)

Hirofumi Abo, MD (Rokkou Hospital)

Tatsuo Akechi, MD, PhD (Nagoya City University Hospitals)

Nobuya Akizuki, MD, PhD (Chiba Cancer Center)

Toru Okuyama, MD, PhD (Nagoya City University Hospital)

Daisuke Fujisawa, MD, PhD (Keio University Hospital)

Shingo Hagiwara, MD (Tsukuba Medical Center Hospital)

Takeshi Hirohashi, MD (Eiju General Hospital)

Takayuki Hisanaga, MD (Tsukuba Medical Center Hospital)

Kengo Imai, MD (Seirei Mikatahara General Hospital)

Shuji Inada, MD, PhD (The University of Tokyo)

Satoshi Inoue, MD (Seirei Mikatahara General Hospital)

Shinichiro Inoue, MD (Okayama University Hospital)

Aio Iwata, MD (National Cancer Center Hospital East)

Akifumi Kumano, MD (Rokkou Hospital)

Takashi Matsui, MD (Tochigi Cancer Center)

Yoshihisa Matsumoto, MD, PhD (National Cancer Center Hospital East)

Naoki Matsuo, MD (Sotoasahikawa Hospital)

Kaya Miyajima, MD, PhD (Keio University Hospital)

Ichiro Mori, MD, PhD (Garcia Hospital)

Sachiyo Morita, MD, PhD (Shiga University of Medical Science Hospital)

Rika Nakahara, MD (National Cancer Center Hospital)

Nobuhisa Nakajima, MD, PhD (Tohoku University Hospital)

Hiroyuki Nobata, MD (National Cancer Center Hospital East)

Takuya Odagiri, MD (Komaki City Hospital)

Ken Shimizu, MD (National Cancer Center Hospital)

Yuki Sumazaki Watanabe, MD (National Cancer Center Hospital East)

Keita Tagami, MD, PhD (Tohoku University School of Medicine)

Emi Takeuchi, MA (Keio University Hospital)

Mari Takeuchi, MD, PhD (Keio University Hospital)

Ryohei Tatara, MD (Osaka City General Hospital)

Akihiro Tokoro, MD (National Hospital Organization Kinki‐Chuo Chest Medical Center)

Megumi Uchida, MD, PhD (Nagoya City University Hospital)

Keiichi Uemura, MD (Hokkaido Medical Center)

Ritsuko Yabuki, MD (Tsukuba Medical Center Hospital)

Naosuke Yokomichi, MD (Seirei Mikatahara General Hospital)
